# Supplementary material for: Effects of combined aerobic and resistance training on glycemic control, blood pressure, inflammation, cardiorespiratory fitness and quality of life in patients with type 2 diabetes and overweight/obesity: a systematic review and meta-analysis
Source: PeerJ. 2024 Jun 14;12:e17525. doi: 10.7717/peerj.17525 (PMC11182026; doi:10.7717/peerj.17525)
Supplement: Supplemental Information 1 [file peerj-12-17525-s001.docx]

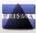
 **PRISMA 2020 Checklist**

|  | **Item** |  | **Reported on Page** |  |
| --- | --- | --- | --- | --- |
| **Section/topic** |  | **Checklist item** |  |  |
|  | **No** |  |  |  |
|  |  |  |  |  |
|  |  |  |  |  |
|  |  |  |  |  |
| **TITLE** |  |  |  |  |
|  |  |  |  |  |
| **Title** | **1** | **Identify the report as a systematic review.** | **In the line number 1** |  |
|  |  |  |  |  |
| **Abstract** |  |  |  |  |
|  |  |  |  |  |
| **Abstract** | **2** | **See the PRISMA 2020 for Abstracts checklist (Table 2).** | **2** |  |
|  |  |  |  |  |
| **INTRODUCTION** |  |  |  |  |
|  |  |  |  |  |
| **Rationale** | **3** | **Describe the rationale for the review in the context of existing knowledge.** | **In the line numbers 95-100** |  |
|  |  |  |  |  |
| **Objectives** | **4** | **Provide an explicit statement of the objective(s) or question(s) the review addresses.** | **In the line numbers 90-100** |  |
|  |  |  |  |  |
| **Methods** |  |  |  |  |
|  |  |  |  |  |
| **Eligibility criteria** | **5** | **Specify the inclusion and exclusion criteria for the review and how studies were grouped for the syntheses.** | **In the line numbers 104 -124** |  |
|  |  |  |  |  |
| **Information** | **6** | **Specify all databases, registers, websites, organisations, reference lists and other sources searched or consulted to** | **In the line numbers 95-100** |  |
| **sources** |  | **identify studies. Specify the date when each source was last searched or consulted.** |  |  |
|  |  |  |  |  |
| **Search strategy** | **7** | **Present the full search strategies for all databases, registers and websites, including any filters and limits used.** | **In the line numbers 95-100** |  |
|  |  |  |  |  |
| **Selection process** | **8** | **Specify the Methods used to decide whether a study met the inclusion criteria of the review, including how many** | **In the line numbers 145- 158** |  |
|  |  | **reviewers screened each record and each report retrieved, whether they worked independently, and if applicable,** |  |  |
|  |  | **details of automation tools used in the process.** |  |  |
|  |  |  |  |  |
| **Data collection** | **9** | **Specify the Methods used to collect data from reports, including how many reviewers collected data from each** | **In the line numbers 145- 158** |  |
| **process** |  | **report, whether they worked independently, any processes for obtaining or confirming data from study investigators,** |  |  |
|  |  | **and if applicable, details of automation tools used in the process.** |  |  |
|  |  |  |  |  |
| **Data items** | **10a** | **List and define all outcomes for which data were sought. Specify whether all Results that were compatible with each** | **In the line numbers 110- 158** |  |
|  |  | **outcome domain in each study were sought (e.g. for all measures, time points, analyses), and if not, the Methods** |  |  |
|  |  | **used to decide which Results to collect.** |  |  |
|  |  |  |  |  |
|  | **10b** | **List and define all other variables for which data were sought (e.g. participant and intervention characteristics,** | **In the line numbers 110- 158** |  |
|  |  | **funding sources). Describe any assumptions made about any missing or unclear information.** |  |  |
|  |  |  |  |  |

**5-1**

| **Study risk of bias** | **11** | **Specify the Methods used to assess risk of bias in the included studies, including details of the tool(s) used,** | **In the line numbers 160- 169**  **And Table S 2** |
| --- | --- | --- | --- |
| **assessment** |  | **how many reviewers assessed each study and whether they worked independently, and if applicable, details of** |  |
|  |  | **automation tools used in the process.** |  |
|  |  |  |  |
| **Effect measures** | **12** | **Specify for each outcome the effect measure(s) (e.g. risk ratio, mean difference) used in the synthesis or presentation** | **In the line numbers 171- 180** |
|  |  | **of Results.** |  |
|  |  |  |  |
| **Synthesis Methods** | **13a** | **Describe the processes used to decide which studies were eligible for each synthesis.** | **In the line numbers 182- 192** |
|  |  |  |  |
|  | **13b** | **Describe any Methods required to prepare the data for presentation or synthesis, such as handling of missing** | **In the line numbers 182- 192** |
|  |  | **summary statistics, or data conversions.** |  |
|  |  |  |  |
|  | **13c** | **Describe any Methods used to tabulate or visually display Results of individual studies and syntheses.** | **In the line numbers 182- 192**  **And Table S4 and S5** |
|  |  |  |  |
|  | **13d** | **Describe any Methods used to synthesize Results and provide a rationale for the choice(s). If meta-analysis was** | **In the line numbers 178- 180** |
|  |  | **performed, describe the model(s), method(s) to identify the presence and extent of statistical heterogeneity, and** | **And table S3** |
|  |  | **software package(s) used.** |  |
|  |  |  |  |
|  | **13e** | **Describe any Methods used to explore possible causes of heterogeneity among study Results.** | **4 and table S2** |
|  |  |  |  |
|  | **13f** | **Describe any sensitivity analyses conducted to assess robustness of the synthesized Results.** | **N/A** |
|  |  |  |  |
| **Reporting bias** | **14** | **Describe any Methods used to assess risk of bias due to missing Results in a synthesis (arising from reporting biases).** | **Line 160 using Revman, and Table S3** |
| **assessment** |  |  |  |
|  |  |  |  |
| **Certainty** | **15** | **Describe any Methods used to assess certainty (or confidence) in the body of evidence for an outcome.** | **Line 178 by GRADEpro**  **And table S3** |
| **assessment** |  |  |  |
|  |  |  |  |
| **Results** |  |  |  |
|  |  |  |  |
| **Study selection** | **16a** | **Describe the Results of the search and selection process, from the number of records identified in the search to the** | **Line 182 and Figure 1** |
|  |  | **number of studies included in the review, ideally using a flow diagram.** |  |
|  |  |  |  |
|  | **16b** | **Cite studies that met many but not all inclusion criteria (‘near-misses’) and explain why they were excluded.** | \| **Table S4**  **And all the reasons were stated** \| \| --- \| \|  \| |
|  |  |  |  |
| **Study** | **17** | **Cite each included study and present its characteristics.** | **Line 194-line 203**  **Also table 1** |
| **characteristics** |  |  |  |
|  |  |  |  |
| **Risk of bias in** | **18** | **Present assessments of risk of bias for each included study.** | **Line 204- line 209 as well as Figure 2 and Figure 3.**  **Also table S2** |
| **studies** |  |  |  |
|  |  |  |  |
| **Results of** | **19** | **For all outcomes, present, for each study: (a) summary statistics for each group (where appropriate) and (b) an effect** | **Table S3** |
| **individual studies** |  | **estimate and its precision (e.g. confidence/credible interval), ideally using structured tables or plots.** |  |
|  |  |  |  |

**5-2**

| **Results of** | **20a** | **For each synthesis, briefly summarise the characteristics and risk of bias among contributing studies.** | **Line 204 – 209 and Figure 2 and Figure 3**  **Also table S2** |  |
| --- | --- | --- | --- | --- |
| **syntheses** |  |  |  |  |
|  | **20b** | **Present Results of all statistical syntheses conducted. If meta-analysis was done, present for each the summary** | **Line 171** |  |
|  |  |  |  |  |
|  |  | **estimate and its precision (e.g. confidence/credible interval) and measures of statistical heterogeneity. If comparing** |  |  |
|  |  | **groups, describe the direction of the effect.** |  |  |
|  |  |  |  |  |
|  | **20c** | **Present Results of all investigations of possible causes of heterogeneity among study Results.** | **table S3** |  |
|  |  |  |  |  |
|  | **20d** | **Present Results of all sensitivity analyses conducted to assess the robustness of the synthesized Results.** | **N/A** |  |
|  |  |  |  |  |
| **Reporting biases** | **21** | **Present assessments of risk of bias due to missing Results (arising from reporting biases) for each synthesis** | **N/A** |  |
|  |  | **assessed.** |  |  |
|  |  |  |  |  |
| **Certainty of** | **22** | **Present assessments of certainty (or confidence) in the body of evidence for each outcome assessed.** | **table S3** |  |
| **evidence** |  |  |  |  |
|  |  |  |  |  |
| **DISCUSSION** |  |  |  |  |
|  |  |  |  |  |
| **Discussion** | **23a** | **Provide a general interpretation of the Results in the context of other evidence.** | **Line 251-261** |  |
|  |  |  |  |  |
|  | **23b** | **Discuss any limitations of the evidence included in the review.** | **Line 386** |  |
|  |  |  |  |  |
|  | **23c** | **Discuss any limitations of the review processes used.** | **Line 386-395** |  |
|  |  |  |  |  |
|  | **23d** | **Discuss implications of the Results for practice, policy, and future research.** | **In the line numbers 370- 384** |  |
|  |  |  |  |  |
| **OTHER INFORMATION** | |  |  |  |
|  |  |  |  |  |
| **Registration and** | **24a** | **Provide registration information for the review, including register name and registration number, or state that the** | **Line 104** |  |
| **protocol** |  | **review was not registered.** |  |  |
|  |  |  |  |  |
|  | **24b** | **Indicate where the review protocol can be accessed, or state that a protocol was not prepared.** | **State protocol not prepared** |  |
|  |  |  |  |  |
|  | **24c** | **Describe and explain any amendments to information provided at registration or in the protocol.** | **N/A** |  |
|  |  |  |  |  |
| **Support** | **25** | **Describe sources of financial or non-financial support for the review, and the role of the funders or sponsors in the** | **NA** |  |
|  |  | **review.** |  |  |
|  |  |  |  |  |
| **Competing** | **26** | **Declare any competing interests of review authors.** | **In the cover letter** |  |
| **interests** |  |  |  |  |
|  |  |  |  |  |
|  |  |  |  |  |
| **Availability of data,** | **27** | **Report which of the following are publicly available and where they can be found: template data collection forms;** | **PeerJ form** |  |
| **code and other** |  | **data extracted from included studies; data used for all analyses; analytic code; any other materials used in the** |  |  |
| **materials** |  | **review.** |  |  |
|  |  |  |  |  |

**5-3**

Article Information: http://dx.doi.org/10.21037/apm-20-2201

*As the checklist was provided upon initial submission, the page number/line number reported may be changed due to copyediting and may not be referable in the published version. In this case, the section/paragraph may be used as an alternative reference.

**5-5** **Updated on September 21, 2020**
