# Supplementary material for: Effects of combined aerobic and resistance training on glycemic control, blood pressure, inflammation, cardiorespiratory fitness and quality of life in patients with type 2 diabetes and overweight/obesity: a systematic review and meta-analysis
Source: PeerJ. 2024 Jun 14;12:e17525. doi: 10.7717/peerj.17525 (PMC11182026; doi:10.7717/peerj.17525)
Supplement: Supplemental Information 2 [file peerj-12-17525-s002.docx]

**Table S1.** Search strategy

| **#** | **Database** | **Algorithm** |
| --- | --- | --- |
| 1 | PubMed | ("Exercise"[Title/Abstract] OR "training"[Title/Abstract]) AND ("diabet*"[Title/Abstract]) |
| 2 | Scopus | Title- ABS (exercise OR training) AND Title- ABS (diabetes) |
| 3 | Google Scholar | allintitle(Exercise OR Training) ( type 2 diabetes) |
| 4 | Cochrane Library | (Exercise OR Training) (type 2 diabetes) |
| 5 | Web of Science | (ALL Exercise OR Training) AND (type 2 diabetes) |
| 6 | Science Direct | (Exercise OR Training) (type 2 diabetes) |
