# Supplementary material for: Effects of combined aerobic and resistance training on glycemic control, blood pressure, inflammation, cardiorespiratory fitness and quality of life in patients with type 2 diabetes and overweight/obesity: a systematic review and meta-analysis
Source: PeerJ. 2024 Jun 14;12:e17525. doi: 10.7717/peerj.17525 (PMC11182026; doi:10.7717/peerj.17525)
Supplement: Supplemental Information 4 [file peerj-12-17525-s004.docx]

**Table S3.** Summary of quality assessment findings (GRADE)**.**

| Outcome | Certainty assessment | | | | | | | N (patients) | | Effect | Certainty |
| --- | --- | --- | --- | --- | --- | --- | --- | --- | --- | --- | --- |
|  | N (studies) | Study design | Risk of bias | Inconsistency | Indirectness | Imprecision | Other considerations | CART | ST | Absolute (95% CI) |  |
| HbAIc | 16 | RCT | serious^a, f^ | not serious | not serious | serious^b^ | none | 384 | 358 | SMD **0.37 lower** (0.6 lower to 0.13 lower) | ⨁⨁◯◯ Low |
| RHR | 2 | RCT | serious^a,^ | serious^e^ | not serious | serious^b^ | none | 32 | 33 | SMD **0.65 lower** (1.59 lower to 0.29 higher) | ⨁◯◯◯ Very low |
| SBP | 10 | RCT | serious^a,g^ | serious^c^ | not serious | serious^b^ | none | 353 | 342 | SMD **0.95 lower** (3.14 lower to 1.24 higher) | ⨁◯◯◯ Very low |
| DBP |  | RCT |  |  |  | Serious^b^ | none | 321 | 305 | SMD **-0.33lower** (-.63lower to -0.04 lower) | ⨁◯◯◯ Very low |
| CRP | 3 | RCT | not serious | not serious | not serious | serious^b^ | none | 95 | 72 | SMD **0.02 lower** (0.32 lower to 0.29 higher) | ⨁⨁⨁◯ Moderate |
| TNF-α | 2 | RCT | not serious | not serious | not serious | serious^b^ | none | 36 | 35 | SMD **0.96 lower** (1.52 lower to 0.39 lower) | ⨁⨁⨁◯ Moderate |
| IL-6 | 2 | RCT | serious^a^ | not serious | not serious | serious^b^ | none | 20 | 20 | SMD **0.94 lower** (1.6 lower to 0.28 lower) | ⨁⨁◯◯ Low |
| CRF | 11 | RCT | serious^a,d^ | serious^c^ | not serious | serious^b^ | none | 209 | 211 | SMD **0.4 higher** (0.02 higher to 0.77 higher) | ⨁◯◯◯ Very low |
| BMI | 16 | RCT | serious^a, f^ | not serious | not serious | serious^b^ | none | 481 | 434 | SMD -**0.19 lower** (-0.57 lower to -0.08 lower) | ⨁⨁◯◯ Low |
| QoL | 3 | RCT | serious^a^ | not serious | not serious | serious^b^ | none | 184 | 174 | SMD **0.29 higher** (0.03 higher to 0.56 higher) | ⨁⨁◯◯ Low |

BMI: body mass index; CI: confidence intervals; *CRF: cardiorespiratory fitness;* *CRP: C-reactive protein; DBP: diastolic blood pressure; HbAIc: glycated hemoglobin; IL-6: interleukin-6;* MD: mean difference; *QoL: quality of life;* RCT: randomized control trials; *RHR: resting heart rate;* SMD: standardized mean difference; *SBP: systolic blood pressure*; *TNF-α: tumor necrosis factor α*

^a^ information regarding the blinding of the participant and the assessor was not provided.

^b^ the included studies recorded a small sample size for both the control and intervention groups.

^c^ there is substantial heterogeneity in the study outcome.

^d^ the study was not randomized in its final form due to the high drop-out rate.

^e^ there is moderate heterogeneity in the involved studies.

^f^ the participants were not blinded.

^g^ the assessors were not blinded.

^h^ there is considerable heterogeneity in the studies.
