# Supplementary material for: Effects of combined aerobic and resistance training on glycemic control, blood pressure, inflammation, cardiorespiratory fitness and quality of life in patients with type 2 diabetes and overweight/obesity: a systematic review and meta-analysis
Source: PeerJ. 2024 Jun 14;12:e17525. doi: 10.7717/peerj.17525 (PMC11182026; doi:10.7717/peerj.17525)
Supplement: Supplemental Information 5 [file peerj-12-17525-s005.docx]

**Table S4.** Excluded full-text articles with reasons.

| **Study** | **Reason** |
| --- | --- |
| [Larose, 2009](#_ENREF_31)  [Jennings et al., 2009](#_ENREF_25) | No related outcome measures |
| [Legaard et al., 2022](#_ENREF_33)  [Bogardus et al., 1984](#_ENREF_7) | Combined diet and exercise intervention |
| [Adamo et al., 2005](#_ENREF_1)  [J Larose, Sigal, Khandwala, & Kenny, 2012](#_ENREF_32)  [Newton Jr et al., 2020](#_ENREF_42)  [Sertbas et al., 2021](#_ENREF_53)  [Tokmakidis, Zois, Volaklis, Kotsa, & Touvra, 2004](#_ENREF_59) | No control group |
| Bello et al. 2014;  [Schreuder, Van Den Munckhof, Poelkens, Hopman, & Thijssen, 2015](#_ENREF_51) | Diabetics and non-diabetic patients were enrolled |
| [Boudou, De Kerviler, Erlich, Vexiau, & Gautier, 2001](#_ENREF_8)  [Kadoglou et al., 2013](#_ENREF_24)  [Wallace, Mills, & Browning, 1997](#_ENREF_62) | The control group performed exercise |
