# Supplementary material for: Effects of combined aerobic and resistance training on glycemic control, blood pressure, inflammation, cardiorespiratory fitness and quality of life in patients with type 2 diabetes and overweight/obesity: a systematic review and meta-analysis
Source: PeerJ. 2024 Jun 14;12:e17525. doi: 10.7717/peerj.17525 (PMC11182026; doi:10.7717/peerj.17525)
Supplement: Supplemental Information 6 [file peerj-12-17525-s006.docx]

**Table S5:** The subsequent reports of original studies.

| Original Studies | Reports of original studies | Reason for inclusion (reported parameters) |
| --- | --- | --- |
| [Sigal et al., 2007](#_ENREF_78) | [Reid et al. 2010](#_ENREF_73) | Quality of life |
| [Church et al., 2010](#_ENREF_25) | [Swift et al. 2012](#_ENREF_80) | HbA1c and CRP |
| [Maiorana et al,. 2001](#_ENREF_60) | Maiorana et al. 2002 | BMI, VO_2_max and RHR |
| [Jorge et al., 2011](#_ENREF_43) | Oliveira et al. 2012 | Blood pressure and VO_2_max |
| Magalhães et al., 2019 | Magalhães et al. 2020 | CRP and TNF-α |
