# Supplementary material for: Effects of combined aerobic and resistance training on glycemic control, blood pressure, inflammation, cardiorespiratory fitness and quality of life in patients with type 2 diabetes and overweight/obesity: a systematic review and meta-analysis
Source: PeerJ. 2024 Jun 14;12:e17525. doi: 10.7717/peerj.17525 (PMC11182026; doi:10.7717/peerj.17525)
Supplement: Supplemental Information 7 [file peerj-12-17525-s007.docx]

1. **The rationale for conducting the systematic review / meta-analysis.**

This review represents the first comprehensive assessment of the efficacy of CART in overweight and obese individuals with T2DM. Incorporating physical activity into managing obesity and T2DM is essential, alongside pharmaceutical treatment, psychological and behavioural therapies, and support for quality of life (QOL). To achieve modest weight loss, reduced body fat, and improved fasting blood glucose levels, it is recommended to engage in 30–60 minutes of moderate-to-vigorous intensity physical activity for most days of the week (Wharton et al. 2020). Our study focuses on overweight/obese individuals with T2DM and aims to examine anthropometric measurements, QOL, cardiovascular health, and biochemical parameters. Obesity is a preventable condition linked to lifestyle, and CART could be a valuable tool for promoting physical activity and body awareness among its practitioners. This study aimed to evaluate the current evidence regarding the effectiveness of CART in treating overweight or obese T2DM patients.

1. **The contribution that it makes to knowledge in light of previously published related reports, including other meta-analyses and systematic reviews.**

This is the first systematic review and meta-analysis conducted to evaluate the effectiveness of CART among overweight and obese T2DM patients. Although some four reviews (systematic review and narrative review), either aerobic or resistant without the combination of these modalities) have examined the effects of aerobic and resistance training among T2DM patients (Grace et al. 2017; Kelley & Kelley 2007; Nery et al. 2017; Yang et al. 2014). But no study has investigated the combined training effect on overweight and obese individuals with T2DM. Our study results from combined aerobic and resistance training on diabetes mellitus offer a holistic approach to management. Integrating both exercise modalities potentially enhance glucose control, insulin sensitivity, and cardiovascular fitness. This combined regimen lead to improved glycemic management and reduced risk factors associated with diabetes, promoting overall health. However, individualising exercise plans and further research are necessary to establish optimal efficacy and safety. Hence, our study shows that combined aerobic and resistance training is a synergistic adjunct to conventional treatment and offers a better outcome.
